# LaPlace's law revisited: Cecal perforation as an unusual presentation of pancreatic carcinoma

**DOI:** 10.1186/1477-7819-5-14

**Published:** 2007-02-02

**Authors:** Kristine D Slam, Sarah Calkins, Frederick D Cason

**Affiliations:** 1 Department of Surgery, University of Toledo, Health Sciences Campus, 3065 Arlington Avenue, Dowling Hall, Toledo, Ohio 43614 USA

## Abstract

**Background:**

Pancreatic cancer is often locally and distally aggressive, but initial presentation as cecal perforation is uncommon.

**Case presentation:**

We describe a patient presenting with pneumoperitoneum, found at initial exploration to have a cecal perforation believed to be secondary to a large cecal adenoma, after palpation of the remainder of the colon revealed hard stool but no distal obstruction. Postoperatively, however, the patient progressed to large bowel obstruction and upon reexploration, a mass could now be delineated, encompassing the splenic flexure, splenic hilum, and distal pancreas. Histological evaluation determined this was locally invasive pancreatic adenocarcinoma, and therefore the true etiology of the original cecal perforation.

**Conclusion:**

Any perforation localized to the cecum must be highly suspicious for a distal obstruction, as dictated by the law of LaPlace.

## Background

The law of LaPlace states: in a long pliable tube, the site of largest diameter requires the least pressure to distend. Hence, in a patient suffering a distal large bowel obstruction, in the setting of a competent ileocecal valve, the cecum is the most common site of perforation. Pancreatic carcinoma is often diagnosed late after aggressive local or distant invasion. However, pancreatic cancer initially presenting with cecal perforation secondary to large bowel obstruction from local colon invasion is distinctly uncommon. We report on the pitfall of missing this diagnosis.

## Case presentation

**Figure 1:**
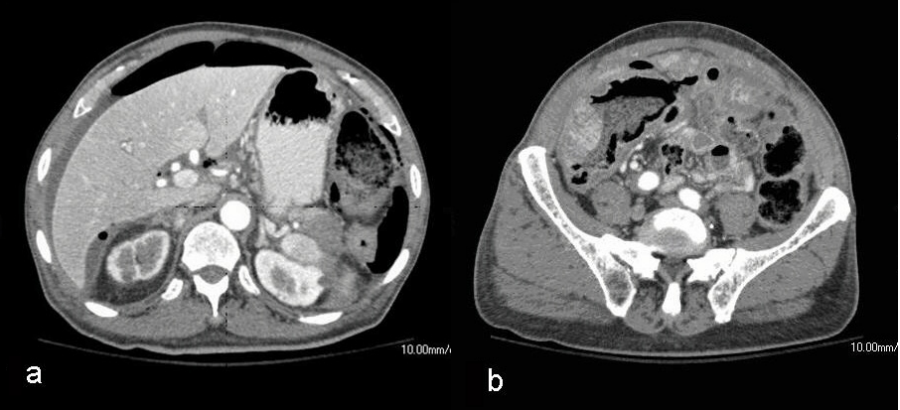
Initial abdominal CT demonstrating free air and fluid and cecal mass; mass in tail of pancreas not initially noted.

A 78 year-old male presented to the emergency department with a ten day history of mild abdominal pain, nausea, and distention, worsening over the last day. He did not recall having a bowel movement for at least three days. His past medical history was significant for hypertension, gout, osteoarthritis, and an eighty-pack year smoking history. He had no prior surgeries and took only a blood pressure medication. He guarded during physical exam, and his abdomen was noted to be quiet, distended, and tender to palpation, but without rigidity or peritoneal signs. His laboratory evaluation was unremarkable. The emergency department obtained a CT scan of his abdomen and pelvis, which demonstrated a large amount of free air and fluid, and a mass could be visualized within the lumen of the cecum. A surgical consultation was emergently obtained (Figure 1).

After fluid resuscitation, he was brought to the operating room for exploration, where we encountered a minimal amount of fecal contamination and perforation of the cecum. A six centimeter non-obstructive mass and a hard mesenteric nodule were found in the cecum just distal to the site of perforation. The remainder of the colon was palpated and noted to be full of hard stool but otherwise without obvious abnormalities. We proceeded with copious irrigation of the peritoneal cavity and performed a right hemicolectomy with primary anastamosis without difficulty.

**Figure 2:**
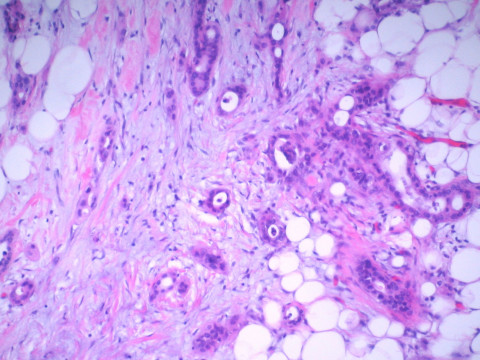
Mesenteric nodule. Invasive malignant glands arising in a dense, fibrotic background. No lymphatic components are visualized.

**Figure 3:**
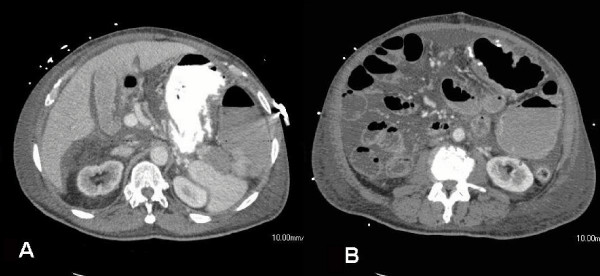
Post operative day six abdominal CT demonstrating pancreatic tail/splenic flexure mass and dilated colon proximal to splenic flexure.

Post-operatively, the patient was extubated but progressed slowly. Final radiologic interpretation of the CT obtained on arrival additionally noted a mass in the tail of the pancreas, a finding the surgical team did not detect on the CT prior to surgery or grossly at exploration. Pathologic evaluation found that the cecal mass contained only tubulovillous adenomatous components and the perforated area demonstrated localized mucosal ischemia but had relatively sharp margins. Well-differentiated metastatic adenocarcinoma was found within the mesenteric nodule, without lymphatic components (Figure 2). The primary tumor responsible for this metastatic nodule was not contained within the surgical specimen. The patient had a slow return of bowel function consistent with ileus, but his abdominal distention increased dramatically overnight on post-operative day five. A repeat CT demonstrated colonic distention proximal to a now apparent mass at the splenic flexure, with distal colon decompression, concerning for a large bowel obstruction (Figure 3). The patient returned to the operating room for reexploration and resection of the obstructive distal colon mass missed at initial operation.

**Figure 4:**
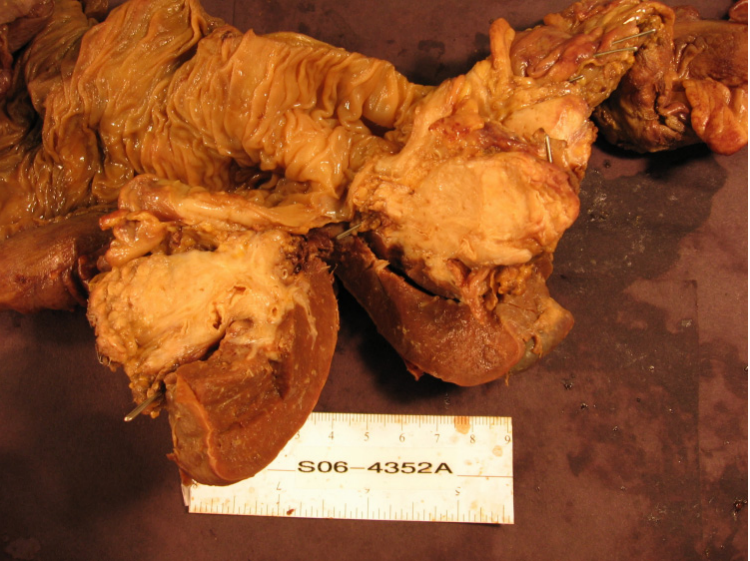
Gross specimen of left colon, pancreas and spleen. The specimen has been sectioned to reveal the mass adjacent to the splenic hilum.

At the second exploration, the entire colon was mobilized, and this time, a mass could clearly be palpated at the splenic flexure of the colon. Continued mobilization revealed that this mass involved the splenic hilum and tail of the pancreas. An en bloc resection of the pancreatic tail, spleen, and left colon was completed without difficulty, and primary anastomosis was completed (Figure 4). The patient was extubated postoperatively and progressed more quickly this time. Final pathologic evaluation of the second specimen was surprising, consistent with mucinous pancreatic adenocarcinoma, extending into the splenic flexure of the colon (T3, N0, M1). At three-month follow up, the patient is doing well living in an extended care facility.

## Discussion

A closed loop bowel obstruction occurs when there is both distal and proximal occlusion to a segment of bowel, resulting in fluid accumulation without passage, strangulating the vascular supply to the affected segment. Typically, a patient with a closed loop obstruction will manifest tachycardia, leukocytosis, fever, or constant pain, but a lack of these symptoms does not exclude the diagnosis [1]. Closed loop large bowel obstructions occur in the presence of a competent ileocecal valve, which inhibits the decompression of colonic fluid and gas into the small bowel [2].

Maintenance of competence at the ileocecal valve involves a complex interaction of anatomic and physiologic properties. The ileocecal valve is composed of two segments, an upper horizonal lip, and a longer and lower concave lip. The longitudinal muscle fibers of the bowel are continuous from the ileum to cecum, and the lips of the valve are formed by the mucous membrane and circular muscle fibers of the intestine [3]. Autopsy studies have documented an additional impact of extrinsic fibrous attachments, the superior and inferior ileocecal ligaments, in maintenance of competence [4]. Finally, manometric testing has demonstrated a tonic pressure at the ileocecal valve that variably responds to bowel distention, nerve stimulation, and pharmacologic manipulation [5]. Barium studies have documented that between 70 and 90% of patients have an incompetent ileocecal valve, placing the approximately 10 to 30% of patients with a competent valve at risk for closed loop large bowel obstructions [6].

Laplace's law dictates that the intraluminal pressure needed to stretch the wall of a hollow tube is inversely proportional to its radius. The cecum is the largest diameter of the colon, and as such, requires the least amount of pressure to distend [7-9]. During a closed loop large bowel obstruction, the wall tension in the cecum increases, causing ischemia to the bowel wall. Microscopically, increasing wall tension can result in a longitudinal splitting of the serosa with a herniation of the mucosa through the diastasis of muscle. On gross inspection, the cecal perforation will typically be found on the anterior longitudinal axis, with sharp uninflammed margins [10].

It has been previously discussed that threshold for increased risk of cecal perforation is a diameter of twelve centimeters [8]. Additional studies have suggested that the duration of dilation may be a more important risk factor for perforation than diameter of the colon [9,11]. The intraluminal pressure required to result in colon perforation has been estimated through colonoscopic studies to be greater than 80 mmHg [12].

Large bowel obstructions distal to the cecum commonly present with proximal colonic dilation, placing the cecum at highest risk for perforation. Possible etiologies of large bowel obstructions include carcinoma, volvulus, fecal impaction, diverticulosis, inflammatory bowel disease, radiation enteritis, or pseudoobstruction [1]. Rarely, reports of pancreatitis result in a closed loop colon obstruction with cecal perforation have been published [13]. The presence of cecal perforation in a previously healthy individual must elicit a suspicion for distal colonic obstruction, especially secondary to carcinoma.

Pancreatic carcinomas lay latent for long periods of time before symptoms develop, determined by tumor location in the pancreas. Carcinomas of the head or uncinate process can cause bile duct, pancreatic duct, or duodenal obstruction. Patients may present with weight loss, painless jaundice, pancreatitis, nausea and vomiting from gastric outlet obstruction, steatorrhea, or back pain. Conversely, tumors of the neck, body, and tail of the pancreas usually do not result in jaundice or gastric outlet obstruction. Often, a mass at this location may only produce vague abdominal pain; new onset diabetes mellitus may be the only symptom of an occult carcinoma [1].

To our knowledge, pancreatic carcinoma initially presenting with local colon invasion, large bowel obstruction, and resultant cecal perforation has not been previously reported. Recent research has focused on the molecular basis for pancreatic carcinoma's aggressive local and systemic spread. Enhanced expression of the cell surface adhesion molecules such as ICAM and VCAM has been demonstrated in pancreatic cancer [14]. Additionally, matrix metalloproteases (MMPs) are transmembrane proteins thought to have significant proteolytic activity on connective tissue in pancreatic carcinoma metastasis [15].

At initial exploration, the incidentally noted cecal mass may have caused us to quickly attribute this as the cause of perforation and inspect the remainder of the colon less thoroughly, missing the true etiology of the large bowel obstruction. At secondary exploration, the gross appearance of the mass suggested colon carcinoma with local pancreatic invasion, so pathologic evaluation with immunohistochemical (CK 7 and CK 20) staining was crucial in obtaining the final diagnosis. The mesenteric mass found at the first operation stained positive for CK7 and negative for CK20, where as the splenic flexure mass was positive for both CK7 and CK20. Colorectal adenocarcinomas are generally CK7 negative, with only 13% positive, and CK20 positive. Approximately 92% of pancreatic carcinomas are positive for CK7 but can be positive or negative for CK20 (Table 1) [16].

**Table 1:** Comparison of colonic carcinoma and pancreatic carcinoma immunohistochemical staining patterns to the malignant tissues of this case adopted from reference [7]

	**CK7**	**CK20**
Colon carcinoma	-	+
Pancreatic carcinoma	+	+/-
Pancreatic-colonic mass	+	+
Mesenteric nodule	+	-

Patients with colorectal cancer that suffer a proximal colon perforation secondary to their cancer have been found to have a lower local recurrence rate and higher disease free survival than those patients suffering a perforation at the tumor site via erosion through bowel wall [17]. Pertaining to pancreatic cancer, only 11% of pancreatic carcinomas are confined to the tail of the pancreas, and over 50% of those pancreatic tail cancers present with stage four disease [18]. Of patients undergoing treatment, the five-year patient survival of a stage four distal pancreatic cancer ranges from 1.6% with radiation alone, to 2.4–2.7% with chemoradiation therapy, to 11.9% with pancreatectomy only, to 19.3% with pancreatectomy plus chemotherapy [18]. However, the survival benefit of an extended en block resection specifically of a locally advanced tail of pancreas cancer is unclear, though some studies have suggested a benefit, especially when combined with neoadjuvant chemoradation treatment [19].

## Conclusion

Distal obstructions of the colon, in the presence of a competent ileocecal valve, may result in colonic perforation. The Law of Laplace dicates that the tension required to distend a hollow tube is lowest at the widest point. Clinically, this explains why the cecum is the most common site of perforation in a distal large bowel obstruction [7-9]. The surgeon must be vigilant at the time of initial exploration for cecal perforation and definitively rule out any distally obstructive cancers. An incidentally found non-obstructive lesion does not rule out a more distally located obstructive process.

## Competing interests

The author(s) declare that they have no competing interests.

## Authors' contributions

**KS **performed literature review, drafted and revised the manuscript

**SC **performed the pathologic evaluation and literature background on the specimen

**FC **originated the idea and revised the manuscript

All authors read and approved the final manuscript.
